# Supplementary material for: Wavelength-scale ptychographic coherent diffractive imaging using a high-order harmonic source
Source: Sci Rep. 2019 Feb 11;9:1735. doi: 10.1038/s41598-019-38501-1 (PMC6370773; doi:10.1038/s41598-019-38501-1)
Supplement: Supplementary file 1 — Supplementary Information [file 41598_2019_38501_MOESM1_ESM.pdf]

Wavelength-scale ptychographic coherent diffractive imaging using a high-order harmonic source

Getnet K. Tadesse[1,2,*], Wilhelm Eschen[1,2], Robert Klas[1,2], Maxim Tschernajew[1,2], Frederik Tuitje[1,3], Michael Steinert[2], Matthias Zilk[2], Vittoria Schuster[2], Michael Zürch[1,3], Thomas Pertsch[2,4], Christian Spielmann [1,3], Jens Limpert[1,2,4] , Jan Rothhardt[1,2]

[1]*Helmholtz-Institute Jena, Fröbelstieg 3, 07743 Jena, Germany*
[2]*Institute of Applied Physics, Abbe Center of Photonics, Friedrich-Schiller-University Jena, Albert-Einstein-Straße 15, 07745 Jena, Germany*
[3]*Institute of Optics and Quantum Electronics, Abbe Center of Photonics, Friedrich-Schiller-University Jena, Max-Wien-Platz 1, 07743 Jena, Germany*
[4]*Fraunhofer Institute for Applied Optics and Precision Engineering, Albert-Einstein-Str. 7, 07745 Jena, Germany*
** Corresponding author: getnet.tadesse@uni-jena.de*

Supplementary Material

Effect of finite spectral width on resolution

In the manuscript, the high resolution reconstruction (Fig. 2(b)) showed differing resolution values along x and y directions due to the differing beam sizes and finite spectral width of the source. To further illustrate this point, reconstructions from measurements that are cropped are shown below. If the diffraction patterns are sufficiently cropped, the effect of finite spectral width will be negligible because they appear at higher diffraction angles. This should then result in lower resolution values that are comparable along x and y directions. In Fig. S1(a), a reconstruction from a measurement cropped to half of the camera size is shown. The largest features of the innermost ring are resolvable both in x and y direction. The cross sections are shown in Fig. S1(b) and show comparable resolution values. This is because features in the diffraction pattern previously blurred due to finite spectral width are not contributing to the current reconstruction. As a result, the resolutions along x and y are comparable here. The second reconstruction is for a measurement that is cropped to a quarter of the camera size (Fig. S1(c)). In this case, the inner ring is not resolved at all while the second innermost ring is clearly resolved in both x and y directions (about 100 nm features). Thus, cropping out parts of the diffraction pattern that are blurred due to finite spectral width of the source results in predictably worse resolution values that are now comparable along x and y directions. This supports the conclusion that the varying resolution of the high resolution measurement in the manuscript (Fig. 2) is indeed caused by the finite spectral width (with differing x and y beam sizes) and not on any artifacts in the reconstruction.

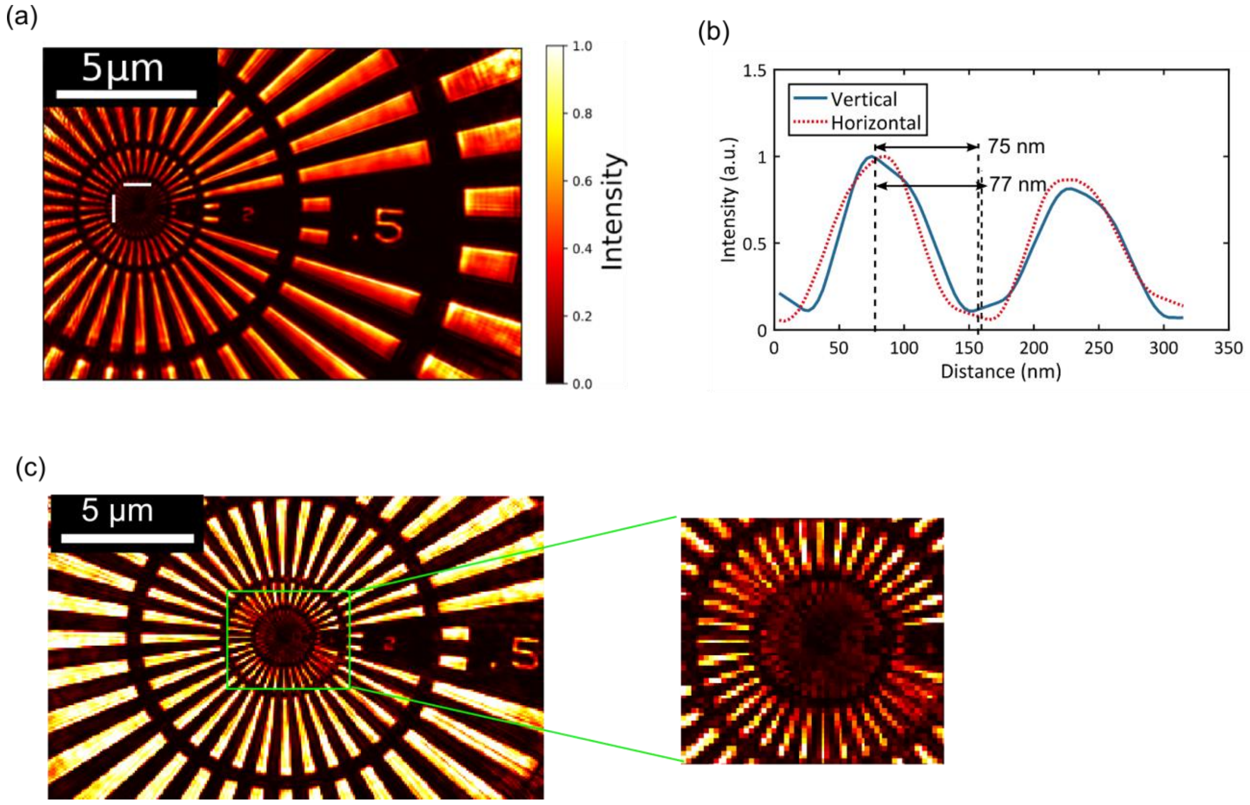

Figure S1 : (a) ptychographic reconstruction of a measurement where the diffraction pattern is cropped to half the camera size (512 × 512 pixels and binning of 2 × 2) (b) cross section along white lines of (a) showing a half pitch resolution of 77 nm along the vertical and 75 nm along the horizontal. (c) reconstruction for a diffraction pattern cropped to a quarter of the camera size (256 × 256 pixels and binning of 2 × 2). The resolution in both x and y is now about 100 nm.

Details of merging diffraction patterns

In the experiment, a standard XUV-Camera (Andor ikon – L) was used to record the diffraction patterns. The detector is limited to a bit-depth of 16 bit which corresponds to roughly 12000 photons at a wavelength of 18 nm considering the camera settings used (Preamp gain of 1, Read-out rate: 1 MHz, High Sensitivity Mode). Since this dynamic range was not sufficient to record photons up to the edge of the detector (see Fig. 1(b)), the dynamic range was increased by measuring two diffraction patterns for every position. One diffraction pattern was recorded with a short exposure time of 1 sec. (Fig. S2(a)) and the second diffraction pattern was recorded with an exposure time of 10 sec. (Fig. S2(b)). To avoid saturation and blooming of the XUV-Camera pixels, a small beam-stop consisting of a 200 µm sphere which was glued to a 15 µm wire was moved right in front of the central speckle in the case of the long exposure measurement. The beam stop was moved in for every position right after the short exposure diffraction pattern was recorded. The short and long exposure measurements were stitched to a single high dynamic

range diffraction pattern (see Fig. S2(c)) by using the central part of the diffraction pattern of the short exposure measurement and the high frequency information of the long exposure measurement. The short exposure diffraction pattern was scaled by a factor of 10 to account for the different exposure times. The shadow of the beam-stop in the long exposure diffraction pattern was estimated from a single diffraction pattern (inset of Fig. S2(b)).

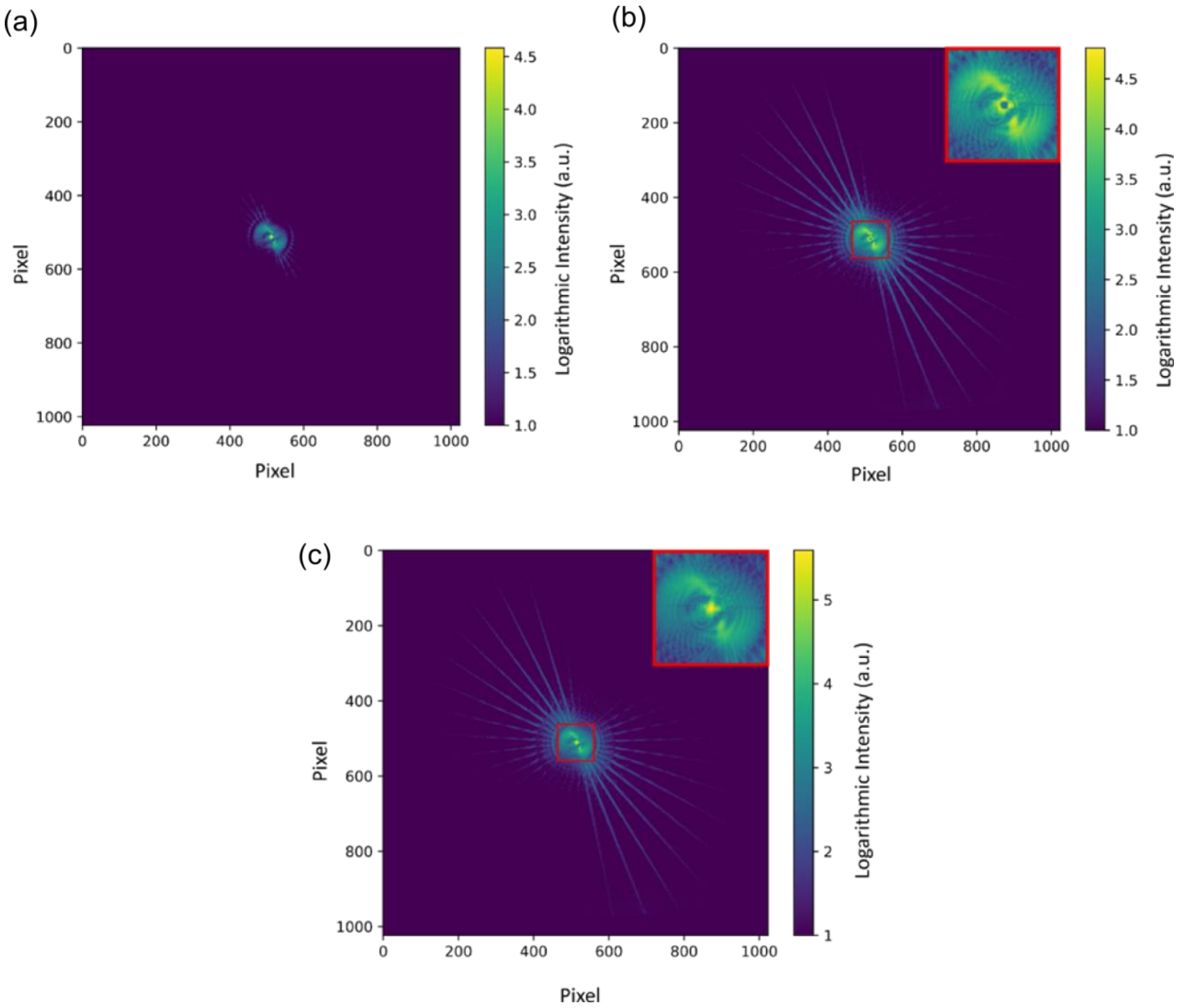

Figure S2 : (a) Diffraction pattern measured with a short acquisition time of 1 second. Only the low spatial frequencies have counts above the noise level. (b) Diffraction pattern measured with a long acquisition time of 10 second. Inset shows the central bright part is now blocked by a beam-stop. (c) a high dynamic range diffraction pattern after replacing the central region of the long acquisition measurement with a scaled version of the short acquisition measurement.

Multi-slice approach

One possible approach for dealing with the fact that the sample is thick is the multi-slice reconstruction proposed by Maiden et al [1]. We performed a comparison of the exit wave profiles from the rigorous finite difference time domain (FDTD) method and the multi-slice technique to determine the accuracy and feasibility of future multi-slice reconstruction. The multi-slice propagation method (part of the multi-slice reconstruction) was proposed by Fleck et al [2] and is also known as fast Fourier transform beam propagation method (FFT-BPM). The 100 nm thick sample is modelled by slices of thickness Δz where the complex transmission function of a slice is computed using the refractive indices of Tantalum at 18.1 nm. For example, 10 slices each with a thickness of 10 nm is one possible model. The method starts by applying a transmission function (amplitude and phase) of the current slice to the incident field with the thin element approximation as

$$E'(x, y, z) = \exp(i[n(x, y, z) - \bar{n}]k_0 \Delta \mathrm{z})\, E(x, y, z)$$

where $\bar{n}$ is the reference refractive index which is that of vacuum, k_0 is the free space wave number, E is the incoming field. The output field E' is then propagated using the angular spectrum method in a homogeneous medium with reference index $\bar{n}$ over a distance of Δz as

$$E(x, y, z + \Delta z) = \mathcal{F}^{-1}\left\{\exp\left(iq\left(k_x, k_y\right)\Delta \mathrm{z}\right) \mathcal{F}\{E'(x, y, z)\}\right\}$$

$$q\left(k_x, k_y\right) = \sqrt{\bar{n}^2 k_0^2 - k_x^2 - k_y^2}$$

Afterwards, the transmission function of the next slice is applied followed by propagation by the angular spectrum method and so on. The transversal grid size was 0.75 nm, the same value as in the FDTD simulation in the manuscript. Periodic boundary conditions were used, but there was enough padding around the structure to make sure that this has no influence. A comparison between the exit waves of the multi-slice (BPM) technique and the FDTD method for a slice thickness of 10 nm is shown in Fig. S3. The x-component of the E-field (the dominant component) of the FDTD result is used for the comparison since the incoming field is x-polarized. It can be seen that the BPM gives an exit wave profile similar to the FDTD result including the waveguiding effect. For a better illustration, the circular cross-section along features of constant size is taken for different half-pitch sizes is shown in Fig. S4. Although the overall profile of the exit waves are similar, there are differences in amplitude between the BPM and FDTD techniques. In addition, the exit wave from FDTD has differing amplitudes for horizontal and vertical features due to the x-polarization of the incoming field. This polarization effect is not resolved in BPM both for the smaller (23 nm) and larger (85 nm) features.

The amplitude and phase errors between the two techniques are shown in Fig. S5 for three slice thicknesses. There is an amplitude error relative to the maximum transmitted amplitude of ± 6% of the 1 nm and 2.5 nm slice thicknesses and ± 8% for the 10 nm slices. The phase errors are mainly in the features outside the etched areas of the Siemen star sample where the field

amplitudes are very small. In conclusion, the multi-slice (BPM) approach can be used to compute the exit wave of the sample but cannot resolve the polarization effects. Since the contrast between the sample's features are strong, the paraxial approximation introduced some errors in the exit field of the multi-slice technique. For samples with weaker sample-probe interaction, the multi-slice approach can give a reasonably accurate exit wave estimate which can be incorporated in the ptychographic reconstruction procedure.

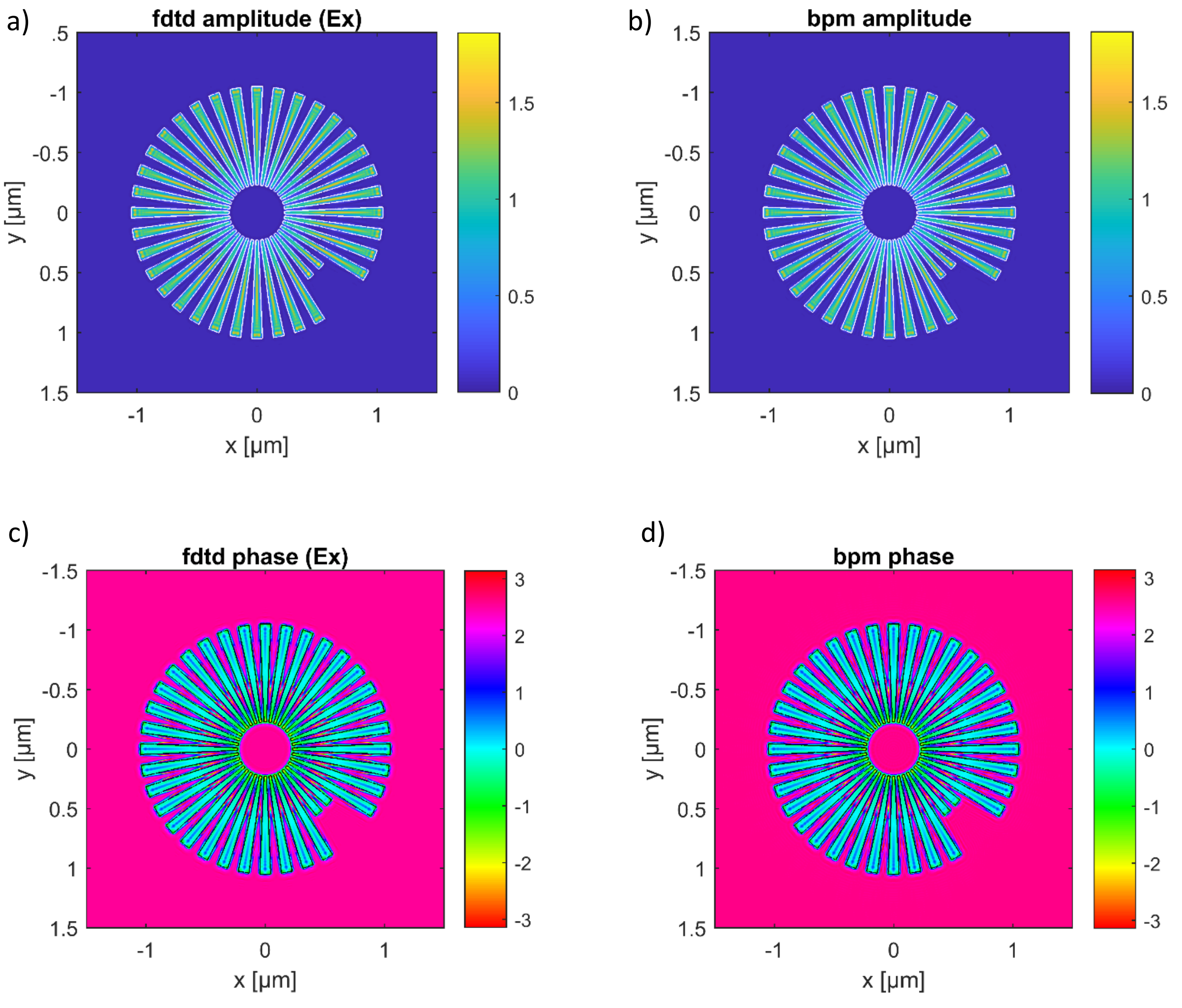

Figure S3 : Comparison of the exit waves of the innermost ring of the Siemens star sample using multi-slice (BPM) and FDTD technique. A slice thickness of 10 nm is used in BPM. (a) and (b) are amplitudes of the exit wave for the FDTD and BPM techniques (color bar is normalized to the incoming field amplitude). (c) and (d) are phases of the exit wave for the FDTD and BPM techniques (color bar is in radians).

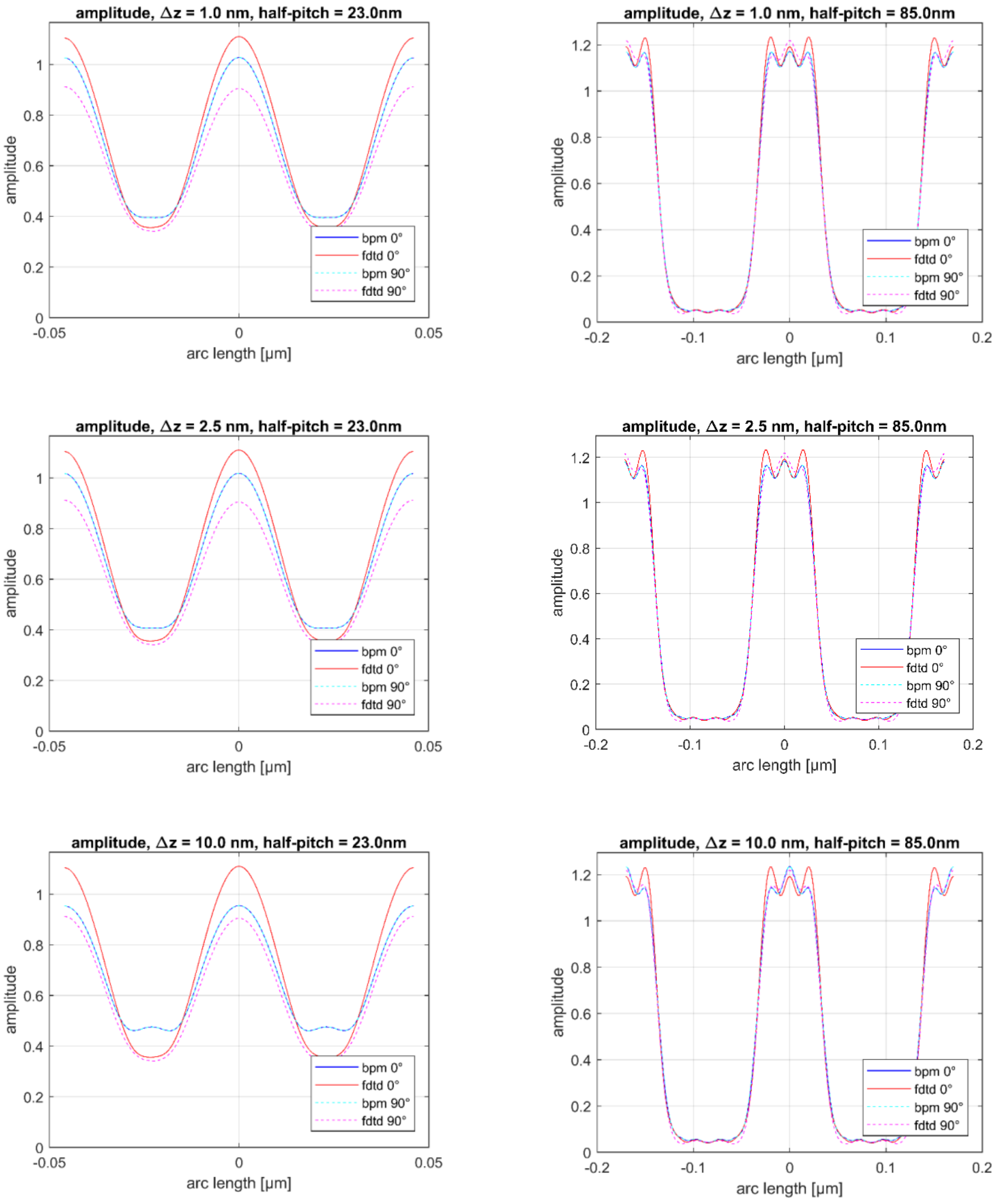

Figure S4 : Comparison of the cross-section of the exit waves of the innermost ring of the Siemens star sample for different slice thicknesses and feature sizes. Angle of 0° in the legend refers to a circular cross-section taken vertically around the x-axis while 90° refers to that taken horizontally around the y-axis. Half-pitch features sizes at the point where the cross-sections are taken are also shown in the figures.

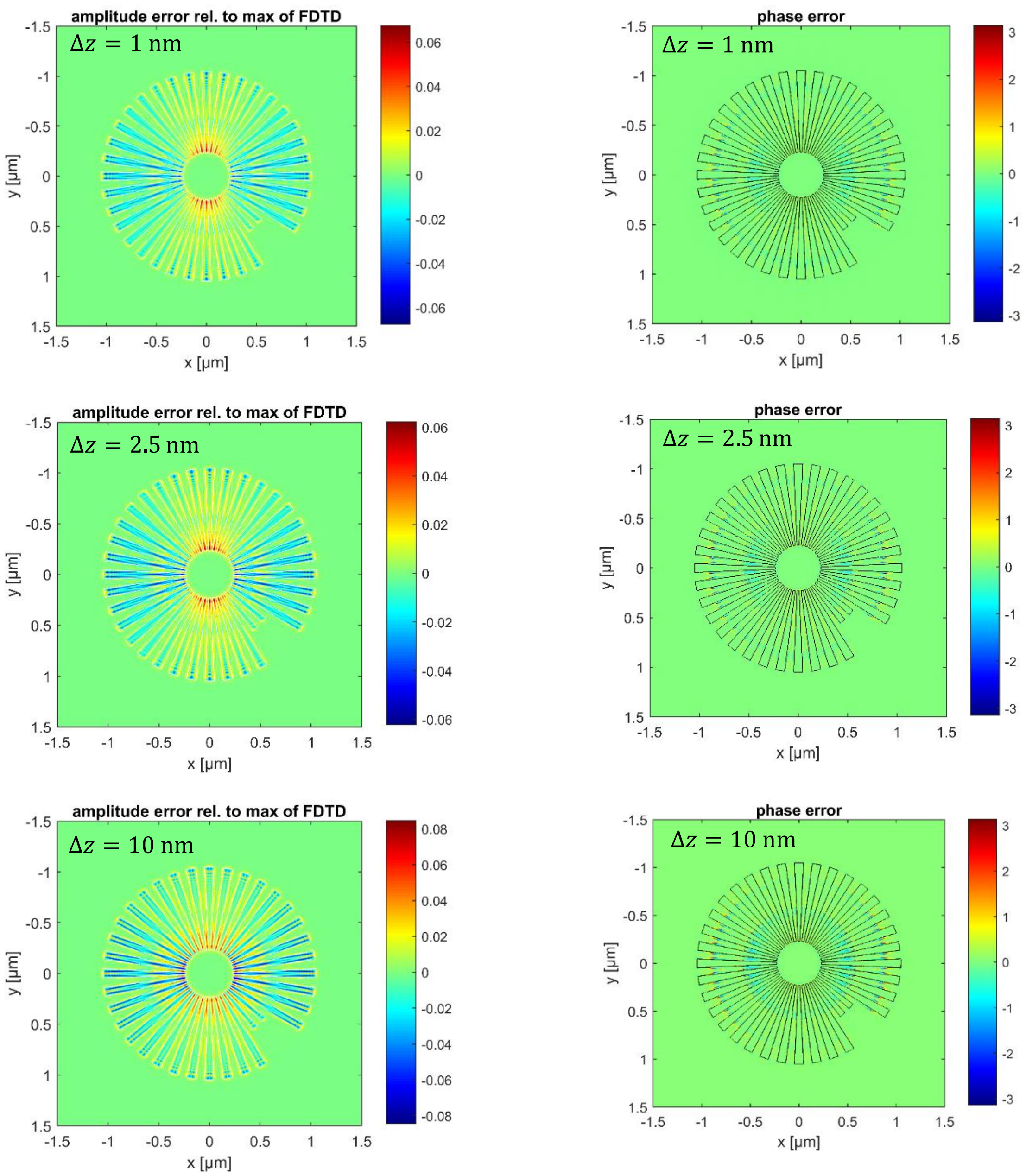

Figure S5 : Amplitude and phase errors of the multi-slice (BPM) approach compared to the FDTD technique. The slice thicknesses used in BPM are shown on the plots. Amplitude error is relative to the maximum of the FDTD amplitude while phase errors are in radians.

References

1. Maiden, A. M., Humphry, M. J. & Rodenburg, J. M. Ptychographic transmission microscopy in three dimensions using a multi-slice approach. *J. Opt. Soc. Am. A* **29,** 1606 (2012).

2. Fleck, J. A., Morris, J. R. & Feit, M. D. Time-dependent propagation of high energy laser beams through the atmosphere. *Appl. Phys.* **10,** 129–160 (1976).
